# Comparative study on the establishment efficacy of four types of animal models of rectovaginal fistula in rabbits

**DOI:** 10.1038/s41598-024-63128-2

**Published:** 2024-05-30

**Authors:** Miaomiao Zhang, Xuhe Zhao, Jianqi Mao, Aihua Shi, Xin Lyu, Yi Lyu, Xiaopeng Yan

**Affiliations:** 1 Department of Hepatobiliary Surgery, The First Affiliated Hospital of Xi’an Jiaotong University, No. 277 West Yanta Road, Xi’an, 710061 Shaanxi China; 2 Shaanxi Provincial Key Laboratory of Magnetic Medicine, The First Affiliated Hospital of Xi’an Jiaotong University, No. 277 West Yanta Road, Xi’an, 710061 Shaanxi China; 3 National Local Joint Engineering Research Center for Precision Surgery & Regenerative Medicine, The First Affiliated Hospital of Xi’an Jiaotong University, No. 277 West Yanta Road, Xi’an, 710061 Shaanxi China; 4 Zonglian College, Xi’an Jiaotong University, Xi’an, China; 5 Department of Pulmonary and Critical Care Medicine, The Second Affiliated Hospital of Xi’an Jiaotong University, No. 3 Shang Qin Road, Xincheng District, Xi’an, 710004 Shaanxi China

**Keywords:** Rectovaginal fistula, Animal models, Rabbits, Gastrointestinal system, Experimental models of disease, Gastrointestinal diseases

## Abstract

Various surgical methods have so far been developed for treating rectovaginal fistula (RVF), each with its own advantages and disadvantages. The lack of standardized animal models of RVF is a major reason for the failure to establish a unified and effective surgical method for the treatment of RVF. This study aimed to explore the feasibility of an RVF animal model by magnetic compression and compare it with the traditional modeling method. Thirty-two female Japanese white rabbits were randomly divided into four groups: A, B, C, and D, based on how the rectovaginal septum was treated. The operation time, intraoperative blood loss, and model success rate of each group were determined. The experimental animals were euthanized 2 weeks after the operation. Their rectovaginal septum specimens were obtained. RVF was observed by the naked eye. The fistula size was measured. Histological changes of fistula were observed by hematoxylin and eosin and Masson staining. All rabbits completed the RVF model and survived 2 weeks after the operation. Groups A and B had no bleeding, while groups C and D had < 0.5 mL of bleeding. The magnet detached in 4–6 days in group A, while it remained in place for 2 weeks after surgery in group B. Only one group D rabbit had a plastic hose for 2 weeks after surgery. The RVFs of groups A and C healed by themselves. In group B, the fistula was well formed. In group D, fistula healing was observed in three animals and the diameter of the fistulas was only 2.82–4.64 mm in the other four animals. Groups B and D had a scar on the inner surface of fistulas. Our study shows that the magnetic compression technique based on the T-shaped magnet is a highly useful method to establishing a continuous and stable RVF model in rabbits.

## Introduction

Rectovaginal fistula (RVF) is a pathological passage between the rectum and the vagina. Its clinical manifestations include vaginal exhaust, defecation, itching, and tingling^1,2^, which adversely affect the quality of life of patients. Some researchers have likened patients with RVF to “dead women walking”^3^. Although RVF is rare, it can be tricky to treat^4^. Various surgical methods, with varying effectiveness, have been developed for treating the RVF^5–8^. However, reoperation after the failure of the first repair of RVF has a significantly reduced success rate, which drops to 55% by the third attempt^9^.

Animal model is a highly useful tool to explore and develop treatment methods of a disease. The pathophysiology of an ideal animal model of a disease should be as similar to human patients as possible. At present, the animal model of RVF is often established by penetrating injury to the rectovaginal septum using a sharp instrument. This method is simple to operate and easy to implement. However, some studies^10^ have shown that the RVF established by this method tends to self-heal. Therefore, some studies have suggested using a plastic tube to indent the rectovaginal septum for creating a fistula^10,11^. In the pre-experimental stage, however, the indwelling plastic tube at the rectal and vaginal septum puncture orifice tends to fall off because of the interference by the experimental animals themselves. Consequently, the stoma could not be maintained.

The magnetic compression technique is the most important component of a magnetic surgical system. The magnetic compression technique can be used to perform vascular anastomosis^12–14^, digestive tract anastomosis^15–18^, ureteral anastomosis^19^, and therapeutic ostomy^20–22^. When the magnetic compression technique was used for digestive tract anastomosis, the compressed tissue is believed to undergo a pathological process of ischemia–necrosis–shedding, while the tissue adjacent to the magnet is thought to undergo adherence–repair–healing to form the anastomosis. A previous study established the stage of digestive tract magnetic anastomosis by using the model of colon magnetic anastomosis in rats^23^. We have also successfully established an animal model of tracheoesophageal fistula in Beagle dogs by using the magnetic compression technique to show pathological changes^24^.

This study investigates the feasibility of establishing an animal model of RVF in rabbits by magnetic compression and compares the results obtained with those achieved using traditional methods to establish a standardized method for preparing animal models of RVF.

## Material and methods

### Ethical statement

We acquired 32 female Japanese white rabbits (age: > 4 months; weight: 3.0–3.5 kg) from the Laboratory Animal Center of the Xi’an Jiaotong University (Xi’an, China) as experimental animals. The experimental protocol was approved by the Committee for Ethics of Animal Experiments of Xi’an Jiaotong University (Permit Number. XJTUAE2023-2209). The research protocol and all the experimental procedures were conducted strictly in accordance with the Guidelines for the Care and Use of Experimental Animals issued by the Xi’an Jiaotong University Medical Center. We ensured animal welfare and minimized animal suffering during the experiment.

### Devices

**Figure 1:**
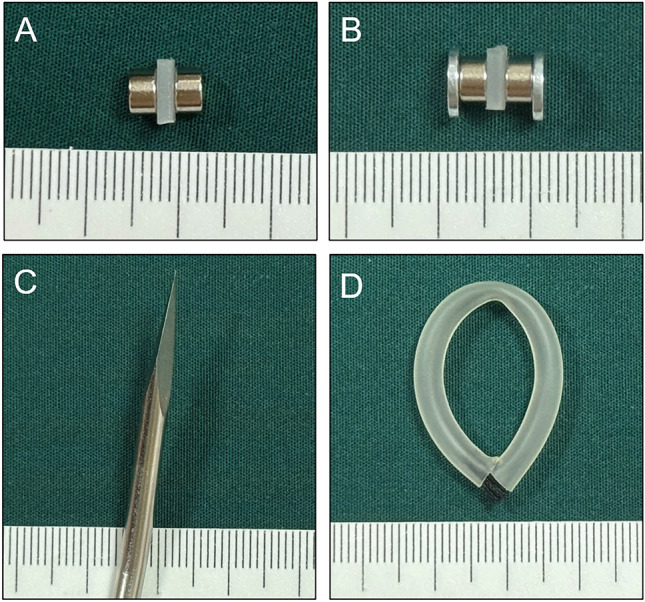
Devices used for the four groups of rabbits. (**A**) Type A magnet; (**B**) type B magnet; (**C**) puncture outfit; (**D**) plastic hose.

We used two types of magnets: type A and type B. The type A magnet is cylindrical, 5 mm in diameter, and 3 mm in height. It is made of N52-sintered NdFeb. It is magnetized in the longitudinal direction and its surface is coated with nickel (Fig. 1A). A single type A magnet weighed 0.44 g. The maximum magnetic field intensity at its compression surfaces is 420 mT. The magnetic force of the two magnets at zero distance can reach 8.26 N. The type B magnet is T-shaped, which is a composite magnet. It comprises 8-mm-diameter and 1-mm-thick NdFeb magnets bonded to the bottom surface of the type A magnet as a base (Fig. 1B). A single type B magnet weighed 0.89 g. The maximum magnetic field intensity at its compression surfaces is 460 mT. The working surfaces of the two magnets had a magnetic force of 10.55 N. A metal rod with an outer diameter of 5 mm was used as the puncture instrument (Fig. 1C). A plastic hose with an outer diameter of 5 mm was used for indenting the rectovaginal septum (Fig. 1D). The details of the devices used in each group are listed in Table 1.

**Table 1:** Devices used in each group and their parameters.

Group	Device	diameter, mm	Height, mm	MFD, mT	Weight, g	Magnetic force**, N
A	Cylindrical magnet	5	3	420	0.44	8.26
B	T-shaped magnet	5/8*	3/1^Δ^	460	0.89	10.55
C	Puncture outfit	5	NA	NA	NA	NA
D	Puncture outfit + plastic hose	5/5^#^	NA	NA	0.54	NA

*Cylindrical magnet diameter/base magnet diameter; ^Δ^cylindrical magnet height/base magnet height; ^#^puncture outfit diameter/plastic hose diameter; ** magnetic force at zero distance.

### Study design

**Figure 2:**
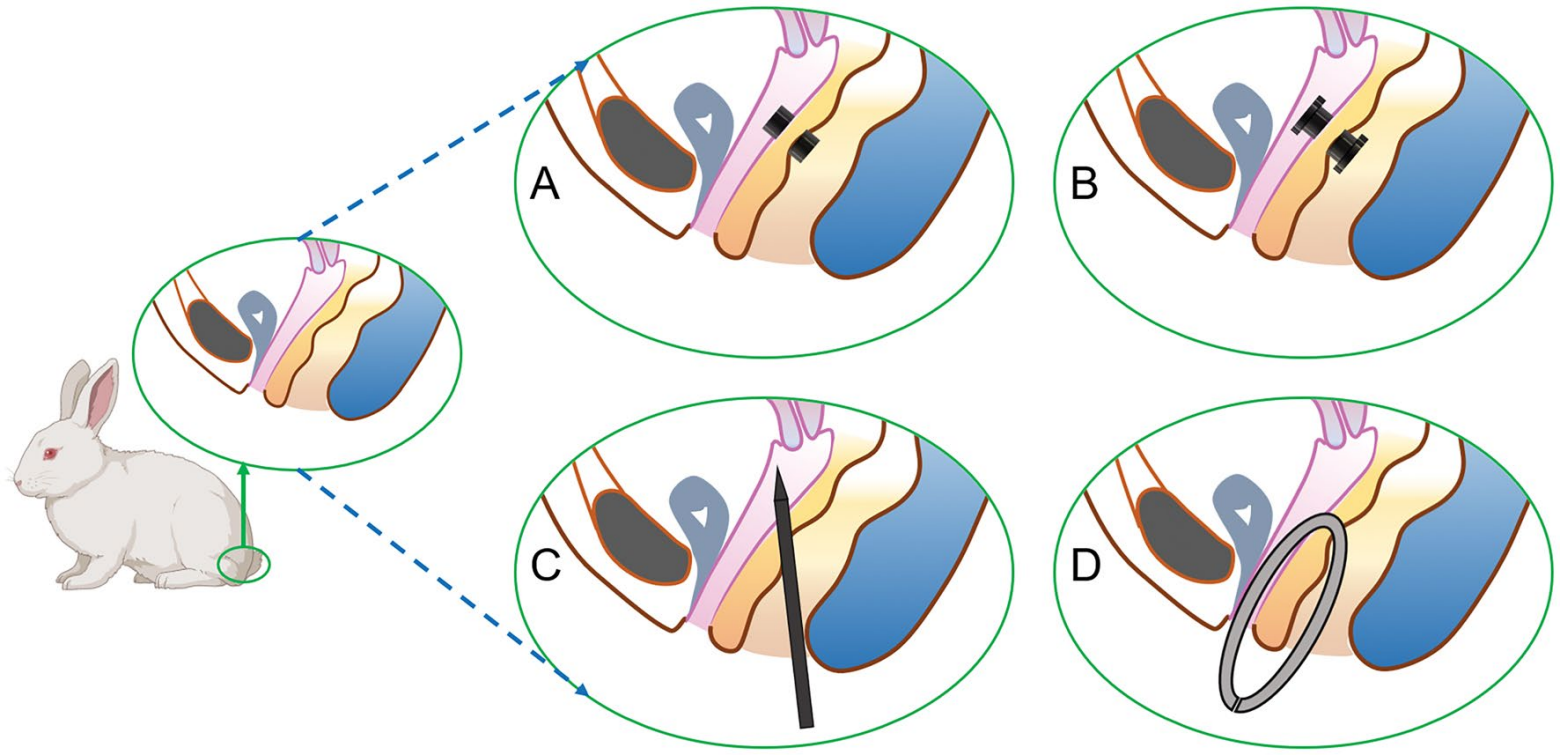
Schematic of the operation of each group. (**A**) Placement of cylindrical magnets in Group A; (**B**) placement of T-shaped magnets in Group B; (**C**) puncturing of the rectovaginal septum in Group C; (**D**) plastic hose inside the rectovaginal fistula (RVF) in Group D.

The 32 experimental rabbits were randomly divided into four groups with eight animals in each group. They were fed adaptively for 1 week after purchase. Then, 3% pentobarbital sodium solution (1 mL/kg) was injected intravenously at the edge of their ears. Once satisfactory anesthesia was achieved, the limbs of the rabbits were fixed in a supine position. The operation diagram of each group is shown in Fig. 2. In group A, two type A magnets were placed on both sides of the rectovaginal septum through the vagina and the anus. These two magnets attracted each other and compressed the rectovaginal septum (Fig. 2A). In group B, T-shaped type B magnets were used. Two T-shaped magnets were placed on each side of the rectovaginal septum through the vagina and the anus. The magnets attracted each other and compressed the rectovaginal septum (Fig. 2B). In group C, the RVF was established by directly penetrating the rectovaginal septum through the anus with a sharp instrument (Fig. 2C). In group D, the RVF was established by conducting a direct anal puncture through the rectovaginal septum with a sharp instrument and then placing a plastic hose in the fistula (Fig. 2D). The operation time and intraoperative blood loss of each group were recorded. The specimens were collected 2 weeks after the operation. The fistula was observed and the diameter of the RVF was measured. The model preparation was considered a success if a stable RVF could be maintained 2 weeks after the surgery and if the variation rate of the fistula diameter did not exceed 20%.

### Postoperative care

All rabbits were managed in a single cage after they emerged from anesthesia. Pethidine hydrochloride (1 mg/kg) was intramuscularly injected every 12 h for 3 days after the operation for analgesia. They were allowed to eat and drink water freely. Their mental state was observed daily. The falling-off time of magnets in groups A and B was recorded and the status of plastic tubes in group D was observed.

### Tissue harvest and analysis

All rabbits were euthanized (3% pentobarbital sodium solution was injected intravenously at 2 mL/kg) 2 weeks after the operation, and their rectovaginal septa specimens were obtained. The RVF was observed by the naked eye and its diameter was measured. The specimens were then soaked overnight in 10% formalin for fixation. Next, the specimens were embedded in paraffin and a 4-μm-thick section from the RVF was prepared. The sections were stained with hematoxylin and eosin (H&E) and Masson trichrome and examined under a bright-field microscope.

### Statistical analysis

SPSS Statistical 20.0 software was used for data analysis. The quantitative data of normal distribution were described by mean ± SD, while those of non-normal distribution were described by the median. Differences between the groups were compared by an independent sample *t*-test or a nonparametric test. *P* < 0.05 indicated a significant difference.

## Results

### Procedural parameters

**Figure 3:**
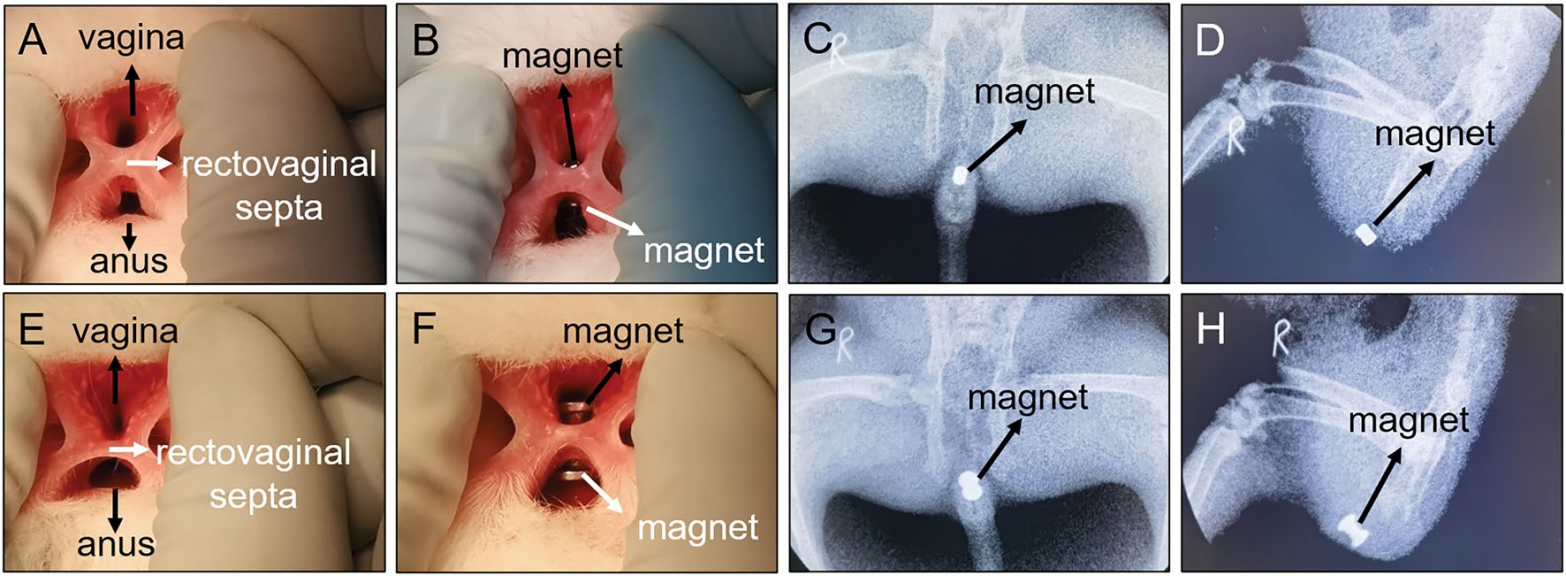
Representative pictures from groups A and B during the procedure. (**A**) The vagina and rectum were exposed; (**B**) type A magnets were successfully placed on the vagina and rectum; (**C**) state of type A magnets as shown in the anteroposterior radiograph; (**D**) state of type A magnets as shown in lateral radiographs; (**D**) exposure of the vagina and anus; (**F**) type B magnets were placed on the vagina and anus; (**G**, **H**) state of type B magnets, as shown in anteroposterior and lateral radiographs.

**Figure 4:**
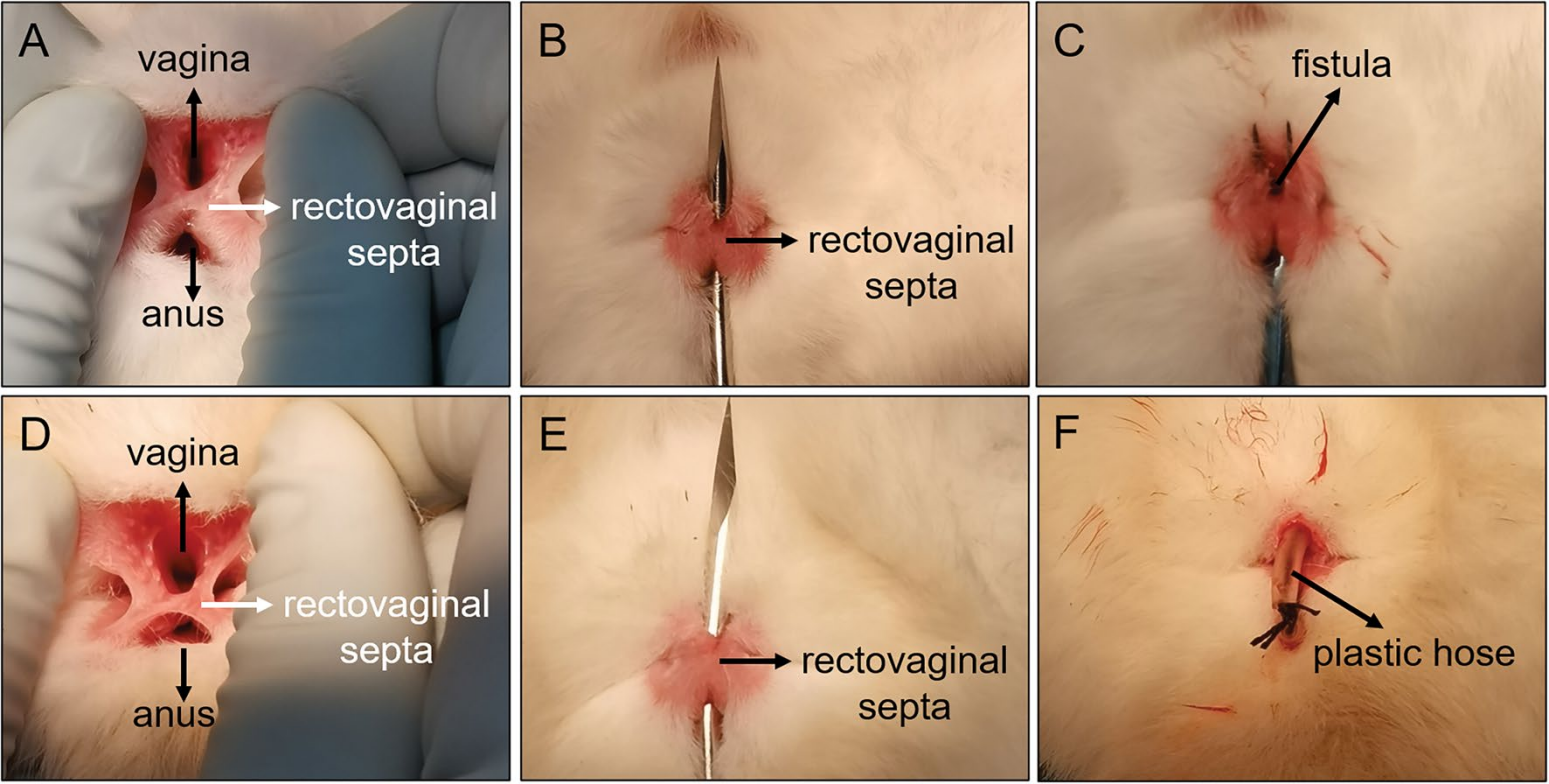
Representative pictures of groups C and D during the procedure. (**A**) The vagina and rectum were exposed; (**B**) the rectovaginal septum was punctured; (**C**) rectovaginal fistula caused by the puncture; (**D**) the vagina and anus were exposed; (**E**) the rectovaginal septum was punctured; (**F**) plastic hose present inside the RVF.

All 32 experimental rabbits successfully emerged from the anesthesia and RVF model operation. All the experimental animals survived to the observation endpoint (2 weeks after operation). The vagina and anus of group A experimental rabbits were exposed and successfully inserted with cylindrical magnets. The two magnets attracted each other and compressed the rectovaginal septum (Fig. 3A, B). An X-ray examination indicated that the magnets were in good position (Fig. 3C, D). No bleeding occurred during the operation. The operation lasted for 36.25 ± 6.94 s (range 30–50 s). In group B rabbits, T-shaped magnets were successfully implanted using the same operation method as that in group A. Here also, the two magnets attracted each other and compressed the rectovaginal septum (Fig. 3E–H). There was no bleeding, and the operation time was 36.88 ± 6.51 s (range 30–50 s). In group C rabbits, a sharp 5-mm-diameter instrument was used to directly puncture the rectovaginal septum through the anus (Fig. 4A–C). Then, the sharp instrument was removed, and the puncture site was compressed by a cotton swab to stop bleeding. Less than 0.5 mL of intraoperative bleeding was observed in group C rabbits. The operation time was 48.13 ± 5.94 s (range 40–55 s). The operation method used in group D rabbits was the same as that used in group C. The sharp instrument was removed and a 5-mm-diameter plastic hose was placed at the puncture site (Fig. 4D–F). Here also, the intraoperative bleeding was less than 0.5 mL. The operation time was 50.63 ± 6.23 s (range 40–60 s). No statistical difference in the operation time between groups A and B (*P*_*A–B*_ = 0.855) or that between groups C and D rabbits (*P*_*C–D*_ = 0.425) was observed. However, statistical differences were noted in the operation time between groups A and B and between groups C and D (*P*_*A–c*_ = 0.002; *P*_*A–D*_ = 0.001; *P*_*B–C*_ = 0.003; *P*_*B–D*_ = 0.001).

### Survival rate and postoperative adverse events

All experimental rabbits were fed in a single cage after their recovery from anesthesia. They were free to eat and drink. Their survival rate was 100% 2 weeks post-surgery. They were generally in good condition after surgery, with no bleeding, serious infection, or rectal obstruction. The cylindrical magnet of the experimental animals in group A was discharged 4–6 days after operation. In contrast, group B rabbits still retained the T-shaped magnets in their rectovaginal septa 2 weeks after surgery. The plastic hose of three group D experimental rabbits fell off on the 5th day after surgery, that of the other three fell off on the 10th day after surgery, and that of one rabbit fell off on the 12th day after surgery. Only one experimental rabbit retained its plastic hose till the end point of the experimental study. The observational data obtained for each experimental group are listed in Table 2.

**Table 2:** Observational data of each experimental group.

Group	Operation time, s^#^	Blood loss, mL	Unplanned shedding, n, %^##^	Fistula diameter, mm^###^
A	36.25 ± 6.94	0	NA	0
B	36.88 ± 6.51	0	0, 0	5.37 ± 0.15
C	48.13 ± 5.94	< 0.5	NA	0
D	50.63 ± 6.23	< 0.5	7, 87.5	2.90 ± 2.94

^#^*P*_*A–B*_ = 0.855; *P*_*C–D*_ = 0.425; *P*_*A–c*_ = 0.002; *P*_*A–D*_ = 0.001; *P*_*B–C*_ = 0.003; *P*_*B–D*_ = 0.001. ^##^According to the experimental design, the magnet of group A was discharged in a planned manner during the observation period. Magnets in group B were not discharged during the observation period. The plastic hose in group D was not discharged during the observation period. ^###^*P*_*B–D*_ = 0.005.

### Gross and histological appearance of anastomosis

**Figure 5:**
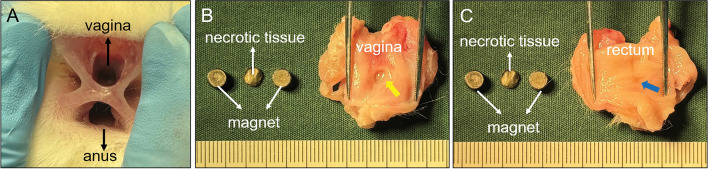
Gross view of rectovaginal septum specimens of Group A. (**A**) Rectovaginal septum was observed in vivo; (**B**) scar of fistula healing can be seen after performing a longitudinal incision on the vagina. (**C**) Scar of fistula healing can be seen after performing a longitudinal incision on the rectum.

**Figure 6:**
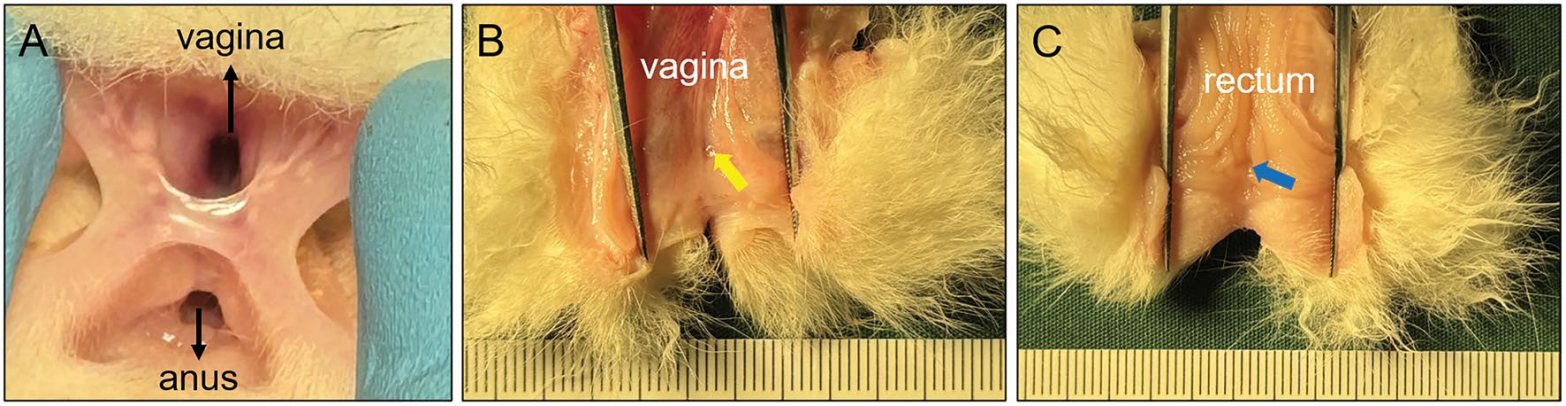
Gross view of rectovaginal septum specimens of Group C. (**A**) Rectovaginal septum was observed in vivo; (**B**) scar of fistula healing can be seen after performing a longitudinal incision on the vagina; (**C**) scar of fistula healing can be seen after performing a longitudinal incision on the rectum.

**Figure 7:**
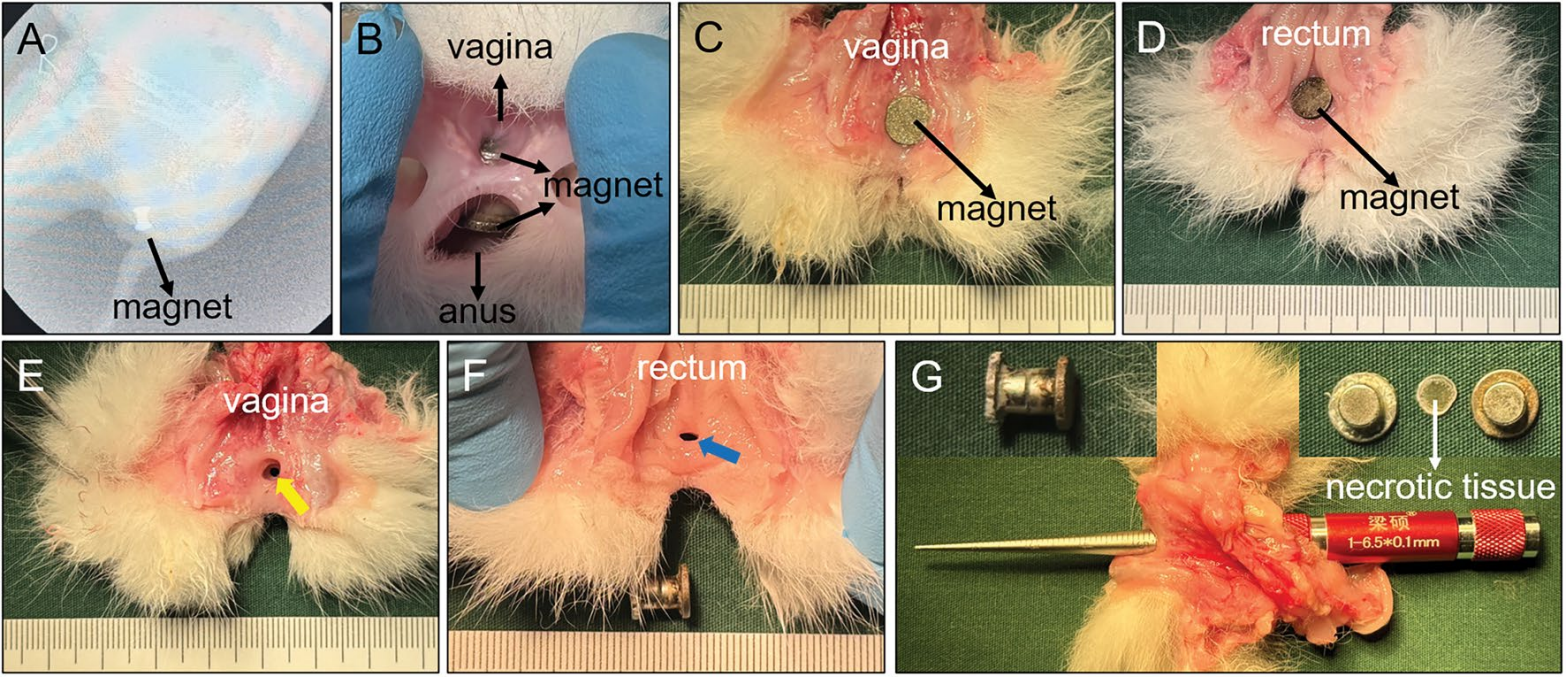
Gross view of rectovaginal septum specimens of Group B. (**A**) X-ray examination 2 weeks after operation showed that the magnet was in good position; (**B**) magnets in the vagina and rectum can be seen in the body; (**C**) longitudinal incision of the vagina shows the magnet located in rectovaginal fistula (RVF); (**D**) longitudinal rectal incision shows the magnet located in RVF; (**E**) RVF was observed on the vaginal side after removing the magnet; (**F**) RVF was observed on the rectum side after removing the magnet; (**G**) T-shaped magnets removed and necrotic tissues shed between magnets. Diameter of the RVF was measured.

**Figure 8:**
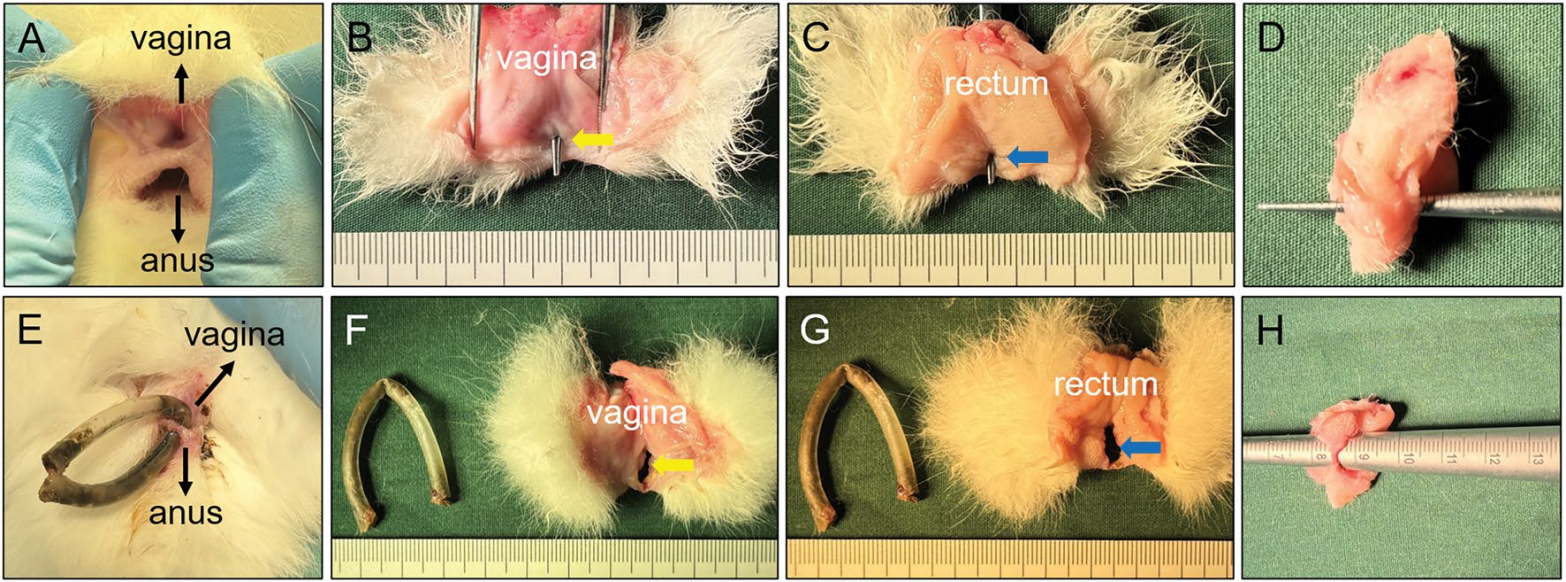
Group D gross specimens of rectovaginal septum. (**A**) Rectovaginal septum of the experimental rabbit with the plastic tube falling off in advance was observed in the body; (**B**) rectovaginal fistula (RVF) as seen after longitudinally dissecting the vagina; (**C**) RVF was observed by longitudinal rectal incision; (**D**) diameter of RVF was measured; (**E**) rectovaginal septum of the experimental rabbit during the retention of the plastic tube; (**F**) RVF was observed by longitudinal incision of the vagina after removing the plastic tube; (**G**) RVF was observed by longitudinal incision of the rectum after removing the plastic tube; (**H**) diameter of the RVF was measured.

**Figure 9:**
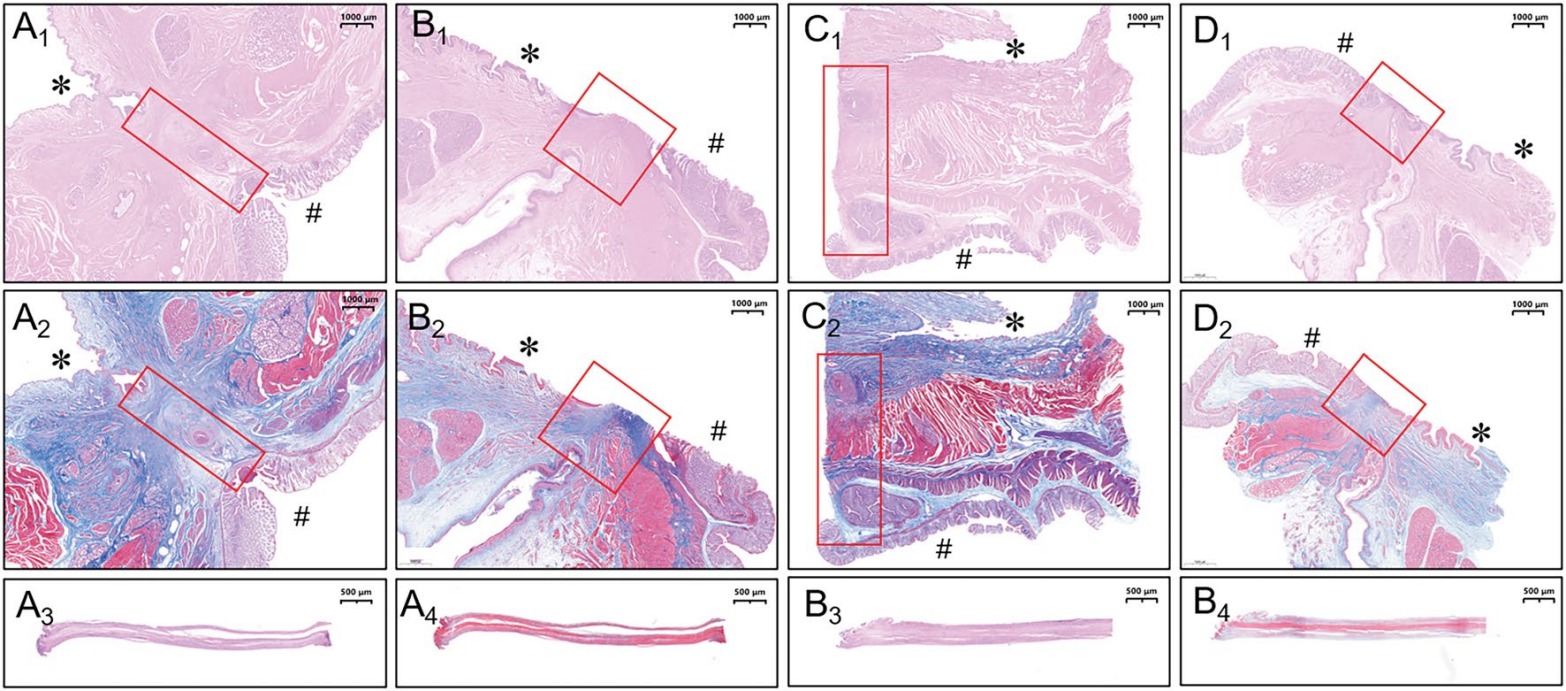
Histological observation of rectovaginal fistula (RVF). (**A**_**1**_) and (**A**_**2**_) H&E and Masson staining of Group A RVF; (**A**_**3**_) and (**A**_**4**_) H&E and Masson staining of necrotic exfoliated rectovaginal septum in Group A; (**B**_**1**_) and (**B**_**2**_) H&E and Masson staining of Group B RVF; (**B**_**3**_) and (**B**_**4**_): H&E and Masson staining of necrotic exfoliated rectovaginal septum in Group B; (**C**_**1**_) and (**C**_**2**_) H&E and Masson staining of Group C RVF; (**D**_**1**_) and (**D**_**2**_) H&E and Masson staining of Group D RVF. (* refers to the vaginal side mucosa; ^#^ refers to the rectal side mucosa; the red box refers to the internal surface of the RVF.)

All 32 experimental rabbits were euthanized 2 weeks after surgery, and gross specimens of their rectovaginal septa were obtained. Their RVFs were observed by the naked eye. The RVFs of group A and C rabbits healed spontaneously 2 weeks after the operation. Scars were observed in the gross specimens after the healing of fistulas (Figs. 5 and 6). The pathologic passage of the rectovaginal septum in group B rabbits was well maintained. The diameter of the RVF was 5.37 ± 0.15 mm (range 5.16–5.60 mm) (Fig. 7A–G). The RVF was healed in three group D rabbits, while their plastic hose fell off on the 5th day. The RVF was also present in four other group D rabbits. Their plastic tube fell off at a later stage. However, they had a significantly reduced fistula with a diameter of 3.69 ± 0.77 mm (range 2.82–4.64 mm) (Fig. 8A–D). The plastic tube did not fall off in only one group D rabbit, although the fistula was torn to the outer margin of the vagina, resulting in a significant enlargement of the fistula (D = 8.48 mm) (Fig. 8E–H). However, such a situation is not suitable for further experiments. Histological observation showed that the healing of the fistula in the groups A and C rabbits was complete, although their rectal mucosa and vaginal mucosa were slightly uneven. In groups B and D, scar formation was observed in the internal lateral tissue of the RVF (Fig. 9).

## Discussion

Only rabbits with the T-shaped magnet provided satisfactory results regarding the preparation of an RVF model, which was beyond our expectation. A careful analysis of surgical procedures and gross specimens of each experimental group showed many major problems that need further discussion.

Previous studies had used rabbits, dogs, and pigs to prepare RVF disease models. They showed that the RVFs of these animals tend to heal on their own^10^. In addition, it was difficult to maintain the stability of the fistula over a long period of time after penetrating the rectovaginal septum and using magnetic compression alone. The fistulas also healed by themselves without any treatment. Therefore, we need to find other animal models that are similar to humans in terms of the RVF. Rabbits have a moderate purchase price and feeding cost. In addition, they are easy to operate and observe after surgery. Therefore, we used rabbits as experimental animal models in this study.

In the present study, we used four groups of rabbits to establish an animal model for RVF. Of these four groups, three had a poor success rate because of the following reasons. First, the end point of observation was set 2 weeks after surgery. Only the rabbits with the T-shaped magnet exhibited satisfactory model effects under such limitations. The RVFs in the rabbits with a cylindrical magnet and those in which a hole was punctured with a sharp instrument healed rather quickly because the fistula was not blocked by foreign bodies. Therefore, if the observation endpoint time is further shortened, these two groups of experimental animals should still have a high success rate. Second, in group D, we passed a plastic tube through the RVF to prevent the fistula opening from healing itself. However, because of the discomfort caused by the indwelling plastic tube, the plastic tube fell out after the animal resisted and interfered. This is a major reason for the poor success rate in group D rabbits, which could be avoided if the rabbits were put on Elizabethan collars after surgery. However, this observation also shows the limitations and inherent shortcomings of the indwelling plastic tube method. For group D, we adopted the surgical operation method reported by Aungst and Roshanravan and obtained similar results^10,11^. However, due to a lack of relevant reports on the preparation of rectovaginal fistula animal models by magnetic compression technique, a comparison with peer research results cannot be conducted. However, our results show that it is easier to prepare the animal model of rectovaginal fistula using the magnetic compression technique. In particular, a higher success rate can be achieved using group B rabbits.

From a histopathological perspective of the RVF, the persistence of a rectovaginal septal fistula is evident from the fibrous scarring or mucosification of the internal surface tissue of the fistula. These tissues take time to change, which explains why the groups A and C rabbits developed fistulas but struggled to maintain them. T-shaped magnets were used in group B rabbits. After the fistula was established, however, it became difficult for the T-shaped magnet to come out by itself because its base was slightly larger than the diameter of the fistula. The retention of the magnet allowed epithelialization or mucosification of the fistula tissue. Notably, because of the unique structure of the T-shaped magnet, it fully entered the rectum and vagina of the animal, without any external parts sticking out of the animal’s body. Consequently, the animal did not resist, which reduced the risk of the magnet falling off. The T-shaped magnet can be kept inside the animal until the researchers began the next step. To remove the magnet, the ends of two vascular forceps were passed through the vagina and the anus to firmly grip the magnet, which were then pulled out to remove the magnet.

Our study showed that the size and location of the fistula can be precisely controlled to improve the consistency of the RVF animal model. However, our study has several limitations too. First, the sample size of experimental animals was quite small. Second, the results would be more convincing if this method could be performed on dogs or pigs. Third, the observation periods were quite long, especially for group B rabbits. Even though it is a limitation, extending the observation time can further help us evaluate the long-term stability of this model-preparation method. In addition, only H&E and Masson staining methods were employed as part of histopathological analysis. We believe that research results would improve if we could identify more indicators through tissue staining.

## Conclusion

We showed that the magnetic compression technique can be used to prepare a rabbit model of RVF. A T-shaped magnet was designed to obtain the optimum model for RVF. We believe that our proposed method can be used as the standard for preparing animal models of RVF.

## Data Availability

The datasets used and analyzed during the current study are available from the corresponding author on reasonable request.
